# Bifurcation in the healing or fibrotic response in a network model of fibrosis: role of the initial injury structure

**DOI:** 10.3389/fnetp.2025.1589216

**Published:** 2025-07-25

**Authors:** Ethan Israel, Joseph K. Hall, Yuqing Deng, Jason H. T. Bates, Béla Suki

**Affiliations:** ^1^ Department of Biomedical Engineering, Boston University, Boston, MA, United States; ^2^ Department of Mechanical Engineering, Boston University, Boston, MA, United States; ^3^ Department of Medicine, University of Vermont, Burlington, VT, United States

**Keywords:** spring network, agent-based model, clustering, healing, percolation

## Abstract

**Introduction:**

Pulmonary fibrosis (PF) is a heterogeneous progressive lung disease characterized by excessive extracellular matrix (ECM) deposition and cross-linking, leading to irreversible tissue stiffening and loss of function. Previous evidence suggests that percolation behavior, where increasing local stiffness facilitates the emergence of stiff regions that span the tissue, underlies the stiffening of the ECM and drives the irreversible mechanical dysfunction. However, it is not fully understood how percolation emerges from the complex interactions between cells and the ECM.

**Methods:**

In this study, we investigated a previously published agent-based spring network model of PF that exhibited bifurcation behavior between healing and fully developed fibrosis as network members were gradually stiffened. By systematically analyzing the configuration of the initial tissue injury, we identify key structural determinants that govern whether an injury heals or transitions into fibrosis.

**Results:**

Results demonstrate that fibrosis is strongly associated with increased initial clustering of injured springs, reduced intercluster distances, and the presence of critical stiffening sites, or hotspots, that act as bifurcation points for disease progression. Furthermore, we show that selectively modifying the stiffness of pivotal network regions at the time of injury can shift the network’s trajectory from fibrosis to healing, highlighting potential intervention targets. These findings suggest that the network structure of tissue injury may serve as a predictive marker for fibrosis susceptibility and provide a mechanistic basis for understanding the nonlinear progression of PF.

## Introduction

Pulmonary fibrosis (PF) is a chronic progressive lung disease characterized by excessive scarring and stiffening of lung tissue, ultimately leading to respiratory failure and death (Nathan et al., 2011). Currently there is no cure, treatment options are limited, and the prognosis is poor, with a median survival time of less than 3 years (Zisman et al., 2005). The activated fibroblasts in the lung play a key role in the progression of PF due to the increased deposition of ECM proteins, which leads to collagen accumulation and ECM stiffening (Martinez et al., 2017; Savin et al., 2022). Although the mechanisms underlying this process are complex and not yet fully understood, PF can be categorized into three stages (Fukuda et al., 1987): 1) the acute stage, characterized by hyaline membrane formation and alveolar damage; 2) the early proliferative stage, marked by prominent interalveolar fibrosis and significant cellular activity; and 3) the remodeled stage, defined by fibrotic and obliterated alveoli with varying airspace sizes. The interaction between signaling pathways and stochastic dynamics determines how the alveolar structure progresses through each stage. For example, cyclic stretch-induced mechanotransduction influences the behavior of fibroblasts by activating the stretch-sensitive ion channels (Murata et al., 2014). In addition, stretch can help release latent TGF-β (Hinz, 2009; Klingberg et al., 2014; Ezzo and Hinz, 2023), which in turn can trigger the transition from fibroblasts to myofibroblasts (Hinz, 2009).

The rich mechanosensitive interactions among the ECM components and the cellular signaling pathways (Tschumperlin et al., 2018) may be studied via a network approach. The general field of network physiology relates to the connectivity and interdependency among physiological systems and their components, and has advanced the understanding of physiology and medicine by analyzing how coupled systems evolve across different stages (Ivanov, 2021). Thus, this can be a useful tool for analyzing the behaviors in PF. For example, an agent-based model on a network was able to explain the formation of the subpleural honeycomb structure in PF (Wellman et al., 2018). Another computational model of PF simulated how profibrotic mediators such as TGF-β1 interact with pulmonary fibroblasts in order to identify potential therapeutic targets (Warsinske et al., 2016). These previous models, however, have not investigated how mechanical stretch and ECM stiffness may affect fibroblast behavior. Therefore, they fail to represent the lung as a dynamic organ, as proposed by network physiology. We recently developed an agent-based spring network model to investigate the dynamic interactions between fibroblasts and their mechanical microenvironment. This model addresses the issue by incorporating a nonuniform network of elastic springs, where migrating agents, representing fibroblasts, can remodel the network by stiffening each member in a stretch- and stiffness-dependent manner (Hall et al., 2024). The outcome of this model showed strong resemblance to histological images of patients with PF. The model also demonstrated a unique feature: as the amount of initial injury increased, the network exhibited a bifurcation behavior whereby the system could self-heal or turn fully fibrotic at a critical level of injury. Furthermore, the model exhibited percolation behavior that leads to different disease phenotypes and outcomes. The implications of such nonlinear dynamic features are of obvious clinical interest because certain patients with the same clinical observation in COVID-related PF may heal (Lorx et al., 2022), whereas others may not, and currently, what determines the final outcome is not understood.

Accordingly, the purpose of the current study was to shed light on the bifurcation behavior of the network model of PF developed by Hall et al. (2024). We hypothesized that the spatial pattern of the initial injury of the spring network is a key determinant of whether an injury can heal or not. We therefore assessed whether the spatial organization of the initial injury could be used to predict the percolation of fibrosis and the ultimate behavior of the network.

## Methods

### An agent-based spring network model

The computational experiments in this study are based on the agent-based spring network model developed by Hall et al. (2024), which simulates the interaction between stiffness and stretch in pulmonary fibrosis. Briefly, the model represents the ECM of lung tissue as a two-dimensional (2D) Voronoi network-based heterogeneous system of interconnected springs, where each spring has its own stiffness ( K ), strain ( ϵ ), and cross-sectional area (CSA). This network is repeatedly solved for equilibrium using simulated annealing (Cavalcante et al., 2005), creating a mechanically responsive structure. Fibroblasts, represented as agents, randomly migrate within the spring network and sense and respond to the local properties of the springs. Specifically, any change in the CSA or ϵ of a spring alters the activation of the agent, which in turn triggers deposition (high activation) or digestion (low activation) of collagen based on the spring’s ϵ and K . This expresses the notion that individual collagen molecules have the same Young’s modulus, but the fiber stiffness depends on how many molecules are in parallel, which in turn determines CSA. Hence, K is proportional to CSA.

### Fibrotic threshold

The agents within the network operate on two physiologically based rules. First, the agents stiffen springs in response to high strain and digest springs in response to low strain; this is a homeostatic negative-feedback system and is responsible for self-healing in the network. Second, the agents stiffen springs in response to high stiffness, which is a positive-feedback system, and it is responsible for fibrosis within the network. Specifically, whenever an agent traverses a spring, it evaluates K and ϵ for that spring, which influence the agent’s activation level. The total activation of an agent ( a ) after traversing a spring is as follows:

$$a=w_{1}a_{\varepsilon }+w_{2}a_{k}-c,$$

where $a_{\varepsilon }$ is agent activation in response to ϵ , $a_{k}$ is agent activation in response to K , and $w_{1}$ and $w_{2}$ are the weighting factors that reflect genetic predispositions or environmental influences toward fibrosis, respectively. The dependence of $a_{\epsilon }$ and $a_{k}$ on ϵ and K is given by two separate Hill functions (Hall et al., 2024). The constant c is determined in such a way that a=0 when the ϵ and CSA are at their homeostatic level. The agent then updates the spring’s CSA as it steps over the spring. As a can be positive or negative, a spring can become stiffer or softer. When $a_{\epsilon }>a_{k}$ and ϵ on the spring is large, the agent attempts to increase CSA and hence K , to reduce ϵ . Alternatively, if $a_{k}>a_{\epsilon }$ , the agent will enter a fibrotic mode to further increase CSA. The complete description of the model together with the parameters used in the simulations can be found in the study by Hall et al. (2024).

In the case of springs with high stiffness, the agent’s response to stiffness dominates the response to strain, and instead of digesting, agents amplify the injury in a positive feedback loop of collagen deposition. The point at which the homeostatic negative feedback turns into positive feedback represents the fibrotic threshold, beyond which the network will not be able to heal from an injury.

### Creating initial network injuries

The homeostatic value of CSA was set to 1. To create an injury at time 0, an initial fibrosis was generated by mimicking the tissue response to some insult such as local inflammation in 20% of the springs within the network by setting their CSA to 5. We tested two different initial injury cases. First, 20% of injured springs were chosen at random. Second, injured springs were localized to a single large cluster to help pinpoint what areas of the injury drove the development of fibrosis. We further evaluated the effects of removing a spring that was deemed to greatly increase the chances of fibrosis.

To simulate disease progression, agents would take a single step across a spring in the network, and then the equilibrium configuration would be solved. This constitutes a single iteration of the simulation. We ran simulations for up to 2,000 iterations, which correspond to the duration of approximately 5 months in real time (Hall et al., 2024; Supplementary Equation S12). To visualize the effects of the injury, each solved network was color-coded to the respective CSAs of the springs to visualize how patterns of stiffness developed and finalized over the progression of fibrosis.

**FIGURE 1:**
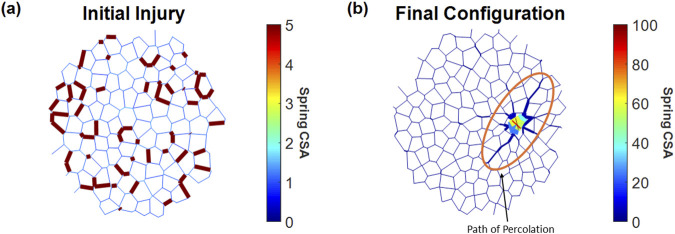
Injury and fibrosis in a 2D spring network. Spring thicknesses are plotted as the logarithm of their relative cross-sectional area (CSA). **(a)** Initial network at the point of injury, where 20% of randomly selected springs were stiffened to a CSA five times their initial value. **(b)** Network with a fibrotic response after 2,000 iterations, showing changes in spring CSA and network topology. Note that the colors represent different CSA values on panels **(a)** and **(b)**.

Figure 1 shows an example of a network with an applied injury at iteration 150 (Figure 1a) and the result of that injury at iteration 2,000 (Figure 1b). In this case, the network was unable to heal, and the injury progressed into fibrosis, which was highlighted by central clustering of stiffened springs and the surrounding affected springs stretched toward the direction of the cluster.

### Geometry of the initial injury

Several methods were used to quantify the geometric configuration of initially injured springs in the network to identify which network configurations result in percolating clusters with a high likelihood of entering an irreversible fibrotic phase transition.

**FIGURE 2:**
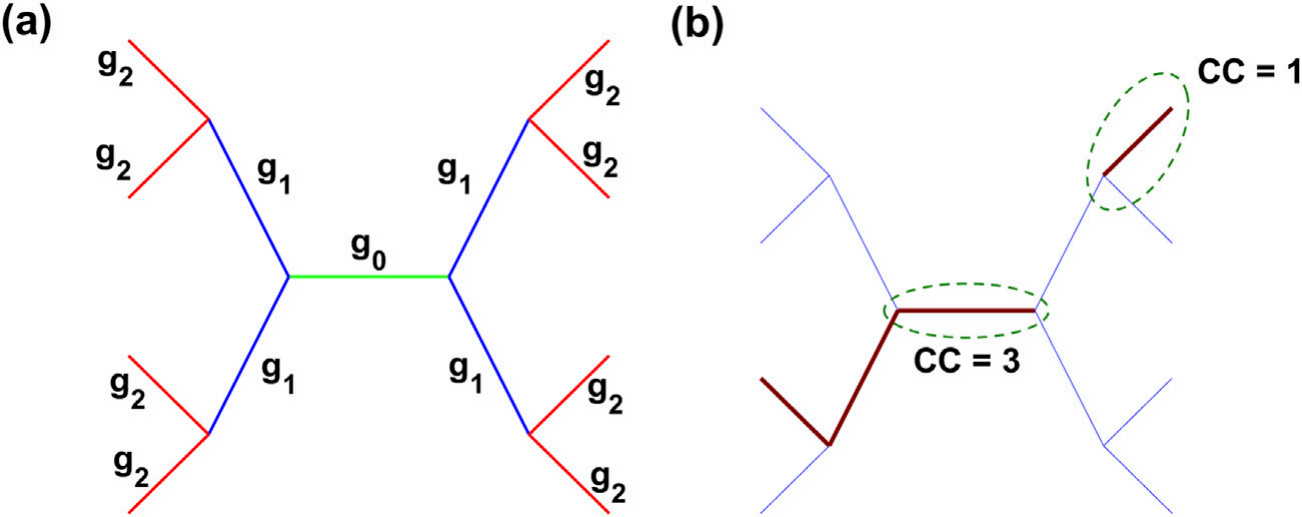
Representation of the clustering coefficient (CC) in a spring network. **(a)** Generations of springs visualized with distinct color coding: generation 0 (green), generation 1 (blue), and generation 2 (red). Labels $g_{0}$ , $g_{1}$ , and $g_{2}$ denote the corresponding generations. **(b)** CC is calculated by counting the number of injured springs (brown) within two generations of the spring of interest. Green dashed ovals highlight example calculations, with CC = 3 for the central spring and CC = 1 for the top-right boundary spring.

The first method involved tracking the number of injured spring neighbors at the initial injury (iteration 150) within a specified proximity to a selected spring. We calculated a clustering coefficient (CC) for each spring in the network as follows. Similar to tree-like data structures, the spring being evaluated was designated the 0th generation. Injured springs connected to the spring being evaluated were designated as first-generation neighbors. Injured springs connected to any first-generation neighbors were designated as second-generation neighbors. To avoid ambiguity in looped topologies common in Voronoi networks, a spring was included in a cluster at generation 2 only if it was connected to generation 0 by an injured spring at generation 1. Figure 2a depicts this branching structure. For example, if a spring and two of its neighbors within two generations are injured, the CC of that spring is 3. Thus, a higher CC indicates greater local clustering of fibrotic injury. Figure 2b illustrates the CC of two clusters, with CC = 3 and CC = 1.

**FIGURE 3:**
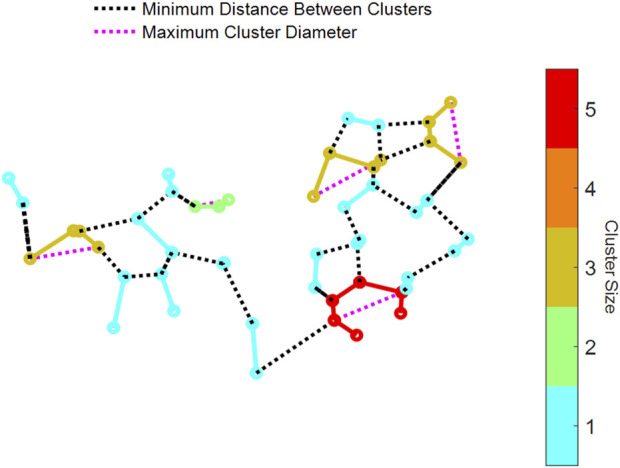
Examples of connected clusters of fibrotic springs in an injured network. Visualization of a network showing clusters that are color-coded by size, as indicated by the color bar on the right. The network includes three key metrics: (1) the minimum Euclidean distance between nodes of one cluster to another cluster (dotted black lines) and (2) the maximum cluster diameter within individual clusters (dotted magenta lines).

To determine how the structure of the initial injury affected the long-term evolution of the network, we determined the clusters of connected injured springs. To do this, we used MATLAB’s sparse() function, which converts a matrix with the nodes of every injured spring into a sparse form. Each nonzero entry in the matrix represents a connection between two nodes. Then, MATLAB’s conncomp() function uses the sparse matrix to determine connected nodes as contiguous clusters and stores the size and nodes of each cluster. Having identified each cluster of injured springs in the network, we evaluated two metrics. First, the Euclidean distance between any two nodes of a cluster was determined (Figure 3), and the largest distance within the cluster was taken as the maximum diameter of the cluster. Second, the minimum distance between each cluster was also computed by calculating the Euclidean distance between the nodes in one cluster and all the nodes in all other clusters (Figure 3).

## Results

Because of the random migration of the agents, the model is fundamentally stochastic in nature. To investigate which statistical parameters related to each injury configuration determine whether a network can heal, 100 different simulations (100 different spring networks, injuries, and network outcomes) were tested, and the properties of the initial injury configurations were analyzed. When 20% of the springs were randomly selected and their CSA was increased from the baseline value of 1 to 5, 43 out of the 100 networks turned fibrotic.

**FIGURE 4:**
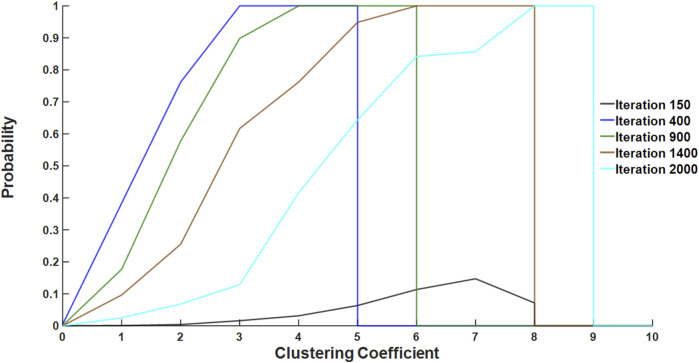
Fibrotic probability as a function of CC for several iteration numbers. Fibrotic probability, calculated as the number of fibrosis-bound springs divided by the total spring count for all simulation data, was plotted over iterations across different CC values. Each colored line represents a specific spread of CC, highlighting geometric differences in the progression of fibrosis. As an example, the probability that a spring with CC of 2 becomes fibrosis-bound is ∼0.8 at iteration 400 but is only ∼0.1 at iteration 2,000.

The number of stiff members in a connected cluster serves as an index of local connectivity, providing insight into whether a region is approaching or has surpassed the percolation threshold, which has a value of ∼0.67 for Voronoi networks in 2D (Becker and Ziff, 2009). To evaluate how regional connectivity changed during the simulations, we analyzed the evolution of the CC of “fibrosis-bound springs,” which are injured springs that turned fibrotic (defined as having a CSA ≥ 5) by 2,000 iterations. The high stiffness of a spring after a long period of healing, 2,000 iterations in this case, indicates a path of irreversible fibrosis. First, the CC of each fibrosis-bound spring was obtained at every iteration, and the total number of springs with a given CC was determined. Then, the fibrotic probability, calculated as the number of fibrosis-bound springs divided by the total number of springs, was obtained as a function of CC and iteration number. These four parameters—CC, total number of springs, number of fibrosis-bound springs, and fibrotic probability—were then calculated for each fibrotic network and summed over all the networks. Figure 4 illustrates the relationship between CC and the fibrotic probability as the simulation progressed. The probability of a fibrosis-bound spring with lower CC occurring decreases as iterations increase, whereas the number of fibrosis-bound springs with higher CC increases with each iteration. This suggests that areas with a higher clustering of springs tend to produce percolation. Indeed, as the simulation progresses, the higher a spring’s CC, the more likely it is to be a fibrosis-bound spring, with a high probability of becoming a part of a developing large fibrotic cluster. This is clearly seen when examining the relationship among iterations 400, 1,200, and 2,000 in Figure 4. As an example, clusters with CC = 5 at iteration 400 may still be developing, and by iteration 2,000, they could be part of a much larger and more connected cluster with CC = 9; this is shown by a lower probability of fibrosis-bound springs with CC = 5 at 2,000 iterations than at 400 iterations.

**FIGURE 5:**
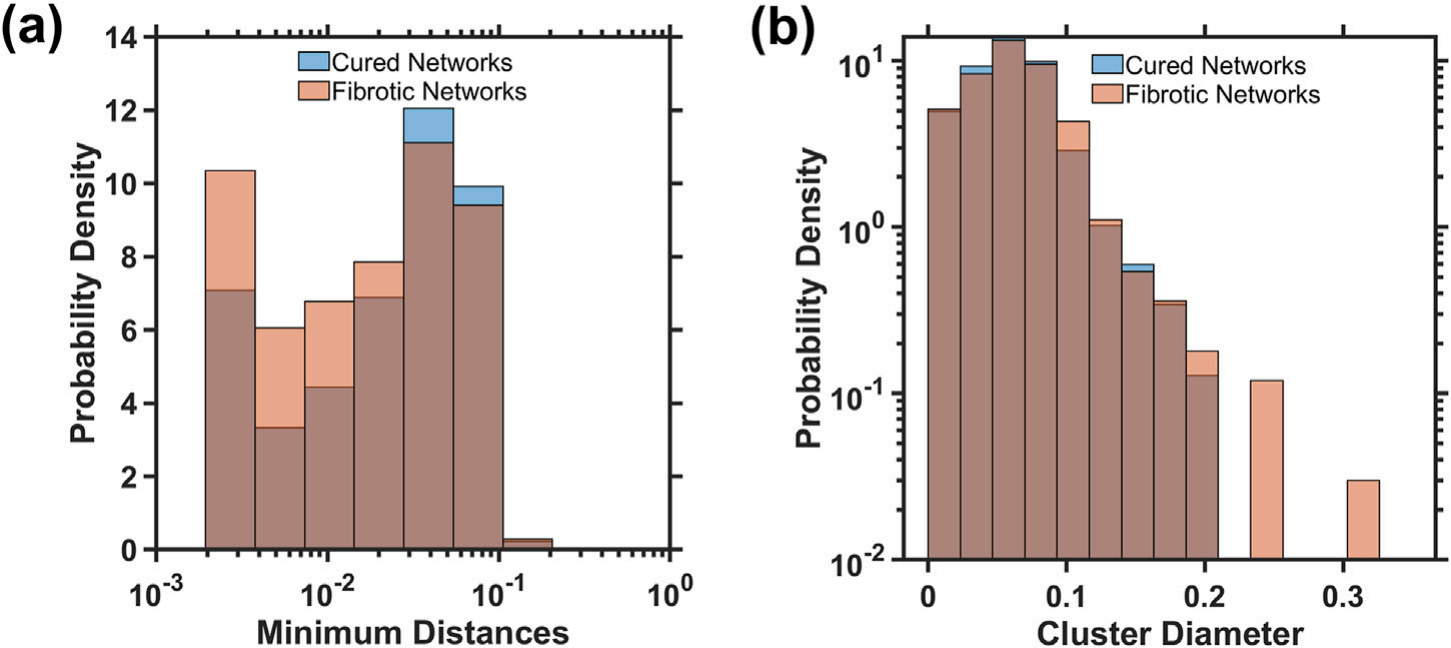
Distributions of the minimum distance between clusters **(a)** and the maximum cluster diameter **(b)** at iteration 150 of the initial injury for both cured and fibrotic networks. The Kolmogorov–Smirnov test found statistically significant differences between the distributions for minimum distance (p < 0.0001) and cluster diameters (p = 0.02).

Figure 5 shows the distributions at the injury of the minimum cluster-to-cluster distance and the cluster diameter measured by the largest internal distance within a cluster for networks that healed or turned fibrotic. The distribution of the minimum distances in fibrotic networks tend to be smaller, suggesting that injured clusters are more densely packed than those in cured networks (Figure 5a). The cluster diameters are larger in fibrotic networks than in cured networks (Figure 5b). A two-sample Kolmogorov–Smirnov test confirms that these differences between fibrotic and cured networks are statistically significant, supporting the hypothesis that smaller distances among the initial fibrotic clusters (p < 0.0001) and larger cluster diameters (p = 0.02) are characteristics of networks developing into fibrosis.

**FIGURE 6:**
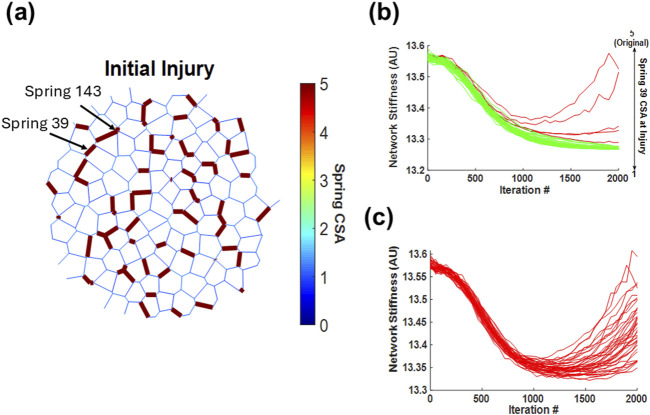
Impact of a single spring’s cross-sectional area on the development of network fibrosis. **(a)** Schematic representation of a network that turns fibrotic, where spring 39 and 143 are part of the initial injury and are the two springs with the highest cross-sectional area by iteration 2,000. **(b)** Trajectories of network stiffness over 2,000 iterations with varied cross-sectional areas of spring 39 at time of injury, illustrating a bifurcation in the network’s response as the stiffness of spring 39 decreases. **(c)** Trajectories for spring 143 across all network simulations, depicting how altering its cross-sectional area has no critical influence on the overall network fibrosis development. In panels **(b)** and **(c)**, red curves indicate simulations where the network ultimately becomes fibrotic, whereas green curves indicate simulations where the network heals.

These results suggest that depending on the initial injury configuration, certain springs are more likely to steer the network toward a fibrotic pathway. To investigate this possibility, we examined a single fibrotic network. The primary contributors to fibrosis were assumed to be (1) the first springs to cross the threshold and enter the positive feedback loop of collagen deposition and (2) the springs with the highest stiffness at the end of the simulation. Springs fitting these criteria usually reside near the center of the cluster and, thus, have the strongest influence on fibrosis progression. Once located, these springs were manually healed to various levels by setting their CSA to a value between 1 and 5 at injury. To do this, the original networks and their injuries were recreated, with the exception of the modified springs, and the network simulations were run again to iteration 2,000. In Figure 6a, the original network’s initial injury configuration highlights spring 39 and spring 143 as the first and second most stiffened springs, respectively, by iteration 2,000. Figures 6b, c show the resulting evolution of the total stiffness of the network as a function of integration of various initial values of CSA. Spring 39 is a critical spring as setting its CSA to 1 at iteration 150 fully prevented the network from turning fibrotic. However, as the initial CSA of spring 39 was gradually increased, the network’s stiffness trajectory diverged from healing to fibrosis at a critical CSA value of 4.3, marking a bifurcation behavior. In contrast, Figure 6c shows that spring 143 is not critical as altering its stiffness at the time of injury had no impact on the final outcome. Furthermore, we observed no apparent correlation between the initial CSA of spring 143 and the final network behavior, as each tested CSA value led to a random final network.

## Discussion

Using a previously published model of pulmonary fibrosis (Hall et al., 2024), we examined the extent to which the spatial configuration of an initial injury determines the long-term outcome of the model. We found that the CC of the initial injury, the size of the clusters, and the spatial arrangement of the clusters all significantly influenced the final state of the network. Furthermore, the model also exhibited a bifurcation behavior similar to that of previously published results: as the stiffness of a single network element within the injury was gradually increased, the system switched from a healing to a fibrotic phenotype.

We expected that regions with high clustering of injured springs were closer to reaching a critical threshold of percolation, resulting in a higher probability of fibrosis. Therefore, we defined the CC for each spring at the time of injury to elucidate the role of connectivity in regions with stiff springs. A high CC suggests a high probability of forming a percolating stiff cluster, which plays a crucial role in the total network stiffness and the corresponding mechanical function (Bates et al., 2007). Evidence of progressive clustering early in fibrosis development is shown in Figure 4. At iteration 400, all fibrotic springs exhibited the highest CC in the network, which was equal to 5. However, by iteration 2,000, the CC increased to 9, indicating that springs with high numbers of stiff neighbors early on in the process persist to become central to percolation and fibrosis progression. Alternatively, low initial CC is expected to lead to healing. Hence, the spatial distribution of the initial injury may differentiate fibrotic networks from those that heal.

For full percolation in the entire network, additional geometric considerations of the initial injury structure may provide insight into whether the network is approaching a critical density of injury. As stiff springs begin to cluster within close Euclidean distances, the density of stiff springs increases, indicating a growing number of interconnected pathways. This clustering effect signifies that the network is approaching a point where a continuous path of stiff springs could span the entire structure, first achieving local and then global percolation, as seen in Figure 1b. Similarly, as the minimum distance between neighboring clusters decreases, a more interconnected and denser stiff network forms, which again moves closer to the threshold where percolation occurs. We expect that a network that has turned fibrotic would, on average, contain larger lengths of initial injury clusters and lower distances between these clusters. Figure 5 suggests that this is indeed the case, as fibrotic networks had lower minimum distances between injured springs and larger lengths of injury than healed networks. These factors provide a metric for the prediction of whether percolation would occur and may also be used to characterize different initial injury structures based on their geometric configuration.

The difficulty in predicting which initial injury leads to fibrosis is also related to the particular agent-based model that we implemented. Specifically, the process of percolation in this model is different from the conventional percolation that fills up a lattice randomly with elements that can cluster and percolate. The model presented in the study by Hall et al. (2024) is an active network in the sense that the agents move around and change their activation depending on their environment. This allows them to carry the information, their activation, to neighboring sites they visit and gradually stiffen. Thus, this behavior is similar to diffusion. A further complication arises from the fact that this diffusion-like process also depends on local strain. If we consider two clusters of stiff springs that are separated by a single soft spring, the strain on this intermediate spring will be high. Consequently, the agents attempt to return strain to the homeostatic level by stiffening the spring. However, this stiffening step may not reduce the strain because the spring bridges two larger stiff clusters that are difficult to stretch. This leads to excessive deposition until the spring’s stiffness exceeds the fibrotic threshold, leading to positive feedback. Hence, two clusters of stiff springs become preferentially connected by a stiff bridge in a near-deterministic manner that cannot happen in regular percolation. This mechanosensitive process in turn accelerates the progression of irreversible fibrosis in a manner reminiscent of the rich-get-richer mechanism (Barabasi and Albert, 1999).

In a dynamical system, bifurcation can be defined as a sudden transition from one type of behavior to a qualitatively different behavior as a model parameter is tuned. Our spring network can be considered a high-dimensional dynamical system with the dimension equal to the number of springs in the network. Figure 6a is an initial condition for the evolution of the system. However, visualizing such a bifurcation is difficult and non-intuitive. Therefore, instead of fully characterizing the bifurcation of the high-dimensional system, we can consider the system as being low-dimensional, with the total network CSA being the dynamical system variable. In this case, the CSA of each spring is a tunable model parameter, and Figure 6b shows that as the CSA of spring 39 is gradually lowered, a new solution, the healed network, becomes possible. In contrast, altering the CSA of spring 143 does not bring about this transition in behavior. Hence, varying the CSA of spring 143 does not cross the boundary of the bifurcation and does not lead to a qualitatively different behavior, that is, healing. To demonstrate this, we consider the following 1D dynamical system:

$$\frac{dK}{dt}=-q\left(K+K_{0}\right),$$

where K is the total stiffness of the network, $K_{0}$ is the baseline stiffness corresponding to a homogeneous network with each spring having a CSA = 1, and q is a structural parameter that is a function of the full network configuration such as that shown in Figure 6a. If the initial condition is $K\left(0\right)=K_{i}$ , the solution of the above equation is as follows:

$$K\left(t\right)=\left(K_{i}-K_{0}\right)e^{-qt}+K_{0}.$$

If q is constructed such that q>0 for all curable configurations, whereas q<0 for all fibrosis-bound configurations, then this system will exhibit a bifurcation from a state of no stable fixed point (red lines in Figure 6b) to a state with a stable fixed point (green lines in Figure 6b).

By examining the fibrotic network in Figure 6a, we determined that the first springs to cross the fibrotic threshold play a central role in steering the network toward fibrosis. The ability to alter the network’s trajectory by modifying just one spring’s initial stiffness at the time of injury demonstrates that divergence of network stiffness is directly initiated by the first spring to enter fibrosis. This finding suggests that within this model, fibrosis progression is not a gradual, distributed process but rather one that is predetermined by the injury configuration and dictated by a single pivotal spring, which may be called a hotspot, through a cascade of events leading to positive feedback, as described above. Indeed, spring 39 is central to a set of seven nearby injured springs separated by several soft springs. When these soft springs become stiffened, suddenly a large, regionally percolating cluster forms in the network, which drives the network toward fully developed fibrosis. However, when the initial injury of spring 39 is lowered below the bifurcation point (4.3), it may be unable to turn the nearby soft springs into fibrotic ones, and the network can heal. Thus, this bifurcation is a result of complex nonlinear network effects providing evidence that the onset of fibrosis—and the subsequent bifurcation between healing and disease—is driven by the earliest structural changes in the network. It therefore appears that the fastest stiffening region may significantly influence the ultimate fate of the tissue, making early intervention at such sites of initial stiffening a potential point of control.

There are two key limitations of this study. The first is that all computational findings come from a single computational model, which constrains the ability to generalize our conclusions to diverse physiological conditions and other modeling frameworks. Nevertheless, this model was developed based on established data from the literature and has been shown to produce highly realistic network structures reminiscent of human pulmonary fibrosis (Hall et al., 2024). Another limitation is that the computational model imposes lung ECM injury instantaneously, whereas in reality, alveolar damage can occur over a time span of 1–2 days. Animal models (Izbicki et al., 2002; Bonatti et al., 2023) and patients following acute lung injury (Zapol et al., 1979) suggest that significant collagen deposition may take several weeks. Additionally, inflammatory cell infiltration, alveolar fluid accumulation, and progressive epithelial cell damage unfold in distinct phases rather than as a single event, emphasizing that some real-world injury processes are more gradual and dynamic (Zemans et al., 2015). Clinically, the initial injury becomes noticeable on CT images only after approximately a year (Snyder et al., 2020), and hence, our modeling results may be relevant to the state of the tissue at the time of diagnosis. The second key limitation is that the network is 2D, whereas the real tissue is 3D. Two important issues arise. First, in 2D, the propagation of mechanical stresses in a random network can be different from that in a regular network (Hall et al., 2023). For our purposes, it is the local strain and the local stiffness that drives the system toward healing or fibrosis. The random nature of the network significantly attenuates stress propagation. Second, the overall behavior is characterized by the overall stiffness of the network, which is primarily determined by whether or not percolation has been reached. The percolation threshold in 3D is significantly lower than that in 2D (Zhukov et al., 2019). These two factors appear to have opposite influences on whether the network heals or becomes fibrotic. Although a bifurcation behavior is expected in 3D, this issue warrants further systematic investigation. Furthermore, our model incorporates separate signaling pathways responsible for how cells respond to stretch and stiffness, whereas in reality, these pathways are not independent (Throm Quinlan et al., 2011). However, it is also known that stiffness, separately from stretch, can induce a fibroblast-to-myofibroblast transition in a positive feedback loop through a COX-2 suppression-related pathway (Liu et al., 2010). Therefore, although the biological responses to these two inputs overlap, to our knowledge, only pathologically high stiffness can trigger pathways that are involved in progressive fibrosis. Finally, we note that there are additional limitations of the model, including the lack of different cell types, lack of airway structures, and the simplistic linear model of ECM mechanics.

The results of the current study provide evidence that pulmonary fibrosis, a heterogeneous disease, can be linked to the ECM’s level of percolation, as quantified by factors that define the connectivity of fibrous tissue. Regions with high connectivity, depending on their stiffness, are more likely to drive the network toward terminal PF. Despite the limitations of the model, the results raise the intriguing possibility that topographic features of fibrotic lesions that can be clinically assessed may be used to predict disease progression in the future.

## Data Availability

The original contributions presented in the study are included in the article; further inquiries can be directed to the corresponding author.
